# Complete mitochondrial genome of *Pseudoechthistatus hei* (Coleoptera: Cerambycidae: Lamiinae) and its phylogenetic analysis

**DOI:** 10.1080/23802359.2022.2145175

**Published:** 2022-11-15

**Authors:** Yu Bai, Lin Ye, Kang Yang, Hui Wang

**Affiliations:** a College of Mathematics and Information Science, Guiyang University, Guiyang City, China; b College of Biology and Environmental Engineering, Guiyang University, Guiyang City, China; c Guizhou Provincial Key Laboratory for Rare Animal and Economic Insects of the Mountainous Region, Guiyang University, Guiyang City, China

**Keywords:** *Pseudoechthistatus hei*, Lamiinae, mitochondrial genome, phylogenetic analysis

## Abstract

The little-known genus *Pseudoechthistatus* Pic, 1917 belongs to the subfamily Lamiinae of the family Cerambycidae. Adult *Pseudoechthistatus hei* Xie and W. Wang, 2019 specimens were collected from Bāijì Hill, Xīntángfáng Village, Wéixī County, Yúnnán Province, China. The complete mitochondrial genome (GenBank accession number: ON641973.1) of *P. hei* was sequenced, annotated, and characterized; it is a circular DNA molecule of 16,103 bp with a 75.71% AT content, and it comprised 13 protein-coding genes (PCG), 22 tRNA genes, two rRNA genes, and 1 control region. The PCGs initiated with the typical ATN (Met) start codons, and were terminated by typical TAN stop codons. The Bayesian Inference phylogenetic tree was first constructed using JTT + F + I + G4 model for *P. hei*, which showed that *P. hei* was closely related to *Monochamus alternatus alternatus*.

## Introduction

**Figure 1.**
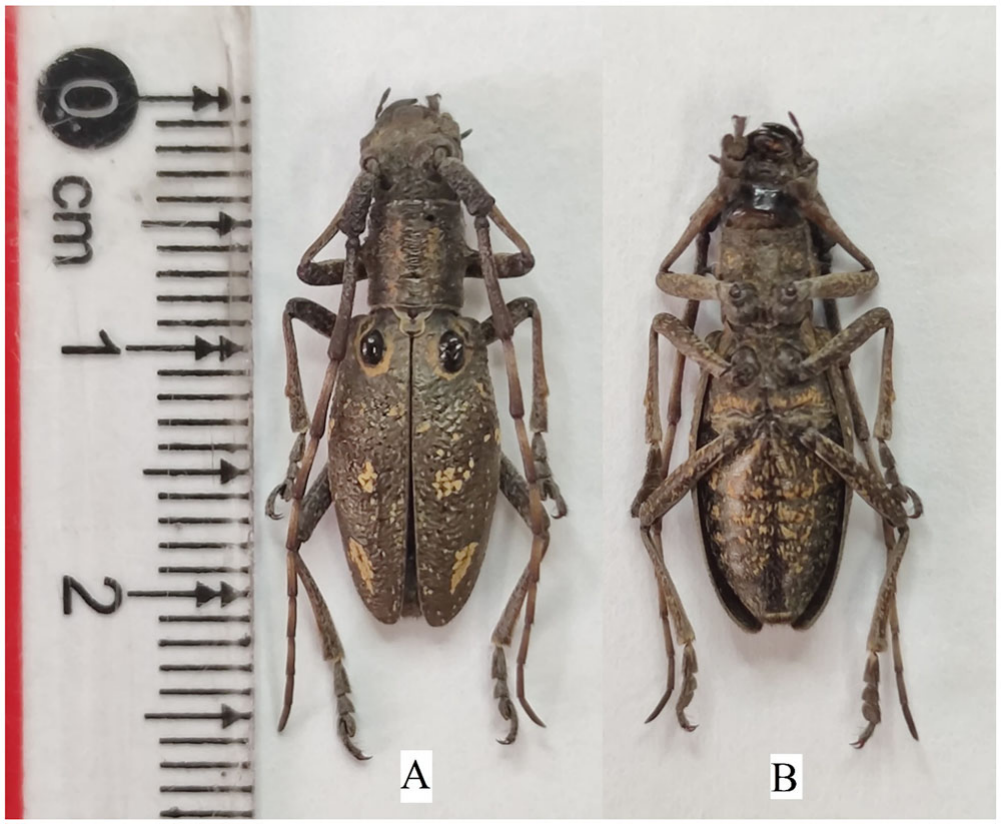
Reference images of adult female of *Pseudoechthistatus hei*. (A) the ventral view of *P. hei*; (B) the dorsal view of *P. hei*. These photographs from our specimens were taken by us. The sex mark including the antennae was about 2.1 times as long as the body of the male while it was 1.3 times as long as the body of the female (Wang P et al. 2019).

The little-known genus *Pseudoechthistatus* Pic, 1917 belongs to the tribe Lamiini of the family Cerambycidae (Bi and Lin 2016), which is unique because of its conspicuously raised sub-basal tubercle on each elytron (Bi and Lin 2016). *Pseudoechthistatus hei* Xie and W. Wang, 2019 has a significantly reduced preapical stripe on the elytron (nearly presenting a very short and narrow longitudinal spot) (Wang P et al. 2019; Figure 1). Currently, the nucleotide database of the National Center for Biotechnology Information (NCBI) contains publicly available information on the mitochondrial genome of the genus *Pseudoechthistatus*. In this study, we first have sequenced and annotated the complete mitochondrial genome (mitogenome) of *P. hei* and constructed phylogenetic trees, which can contribute to the understanding of its mitogenome characteristics and determination of the phylogenetic position of the genus *Pseudoechthistatus*.

## Materials

Adult *P. hei* specimens were collected from Bāijì Hill (99.22° N, 27.42° E), Xīntángfáng Village, Wéixī County, Yúnnán Province, China, on May 21, 2021, and deposited in the animal specimen room of Guiyang University (http://en.gyu.cn/, Yu Bai, dk0001@gyu.edu.cn) under the voucher number GYU-20210521-001.

## Methods

Genomic DNA was isolated using the Qiagen DNeasy Blood and Tissue Extraction kit (Qiagen, Germantown, MD, USA) and subjected to paired-end sequencing (2 × 150 bp) of 300 bp inserts using an Illumina NovaSeq 6000 platform (Illumina, Inc., San Diego, CA, USA). The raw reads were filtered using fastp v0.23.2 (https://github.com/OpenGene/fastp) (Chen et al. 2018). Quality control (QC) standards of reads from DNA were as follows: (1) trimming adapter sequences with >6 bases, (2) removing reads with >0 unidentified nucleotides (N), (3) removing reads with >20% bases with Phred quality < Q30, and (4) removing reads with <150 bases. The genome was assembled *de novo* using NOVOPlasty v4.3.1 (https://github.com/ndierckx/NOVOPlasty) (Dierckxsens et al. 2017) with default parameters and the mitogenome of *Monochamus sparsutus* (GenBank accession number: MW067124) as a seed sequence. The AT-skew [(A − T)/(A + T)] and GC-skew [(G − C)/(G + C)] of the sequence were estimated to investigate the nucleotide composition bias using Perna and Kocher’s formula (Perna and Kocher 1995). The *P. hei* mitogenome was initially annotated using GeSeq Version 2.03 (https://chlorobox.mpimp-golm.mpg.de/geseq.html) (Tillich et al. 2017), using the third-party software tRNAscan-SE v2.0.7 (Chan and Lowe 2019), ARWEN v1.2.3 (Laslett and Canbäck 2008), and BLAT v36x7 (Kent 2002) with the mitogenome of *Monochamus sparsutus* as a reference. The start and stop codons of the protein-coding genes (PCG) were corrected manually using the mitogenomes of *M. sparsutus*, *M. alternatus alternatus* (MT547196) (Liao et al. 2020) and *M. alternatus* (KJ809086) (Li F et al. 2016) as references. The order and orientation of the genes were determined and plotted using the OGDRAW web server (https://chlorobox.mpimp-golm.mpg.de/OGDraw.html) (Greiner et al. 2019). For phylogenetic analyses, the mitogenomes of 19 Lamiinae species and two outgroup species were used to construct a Bayesian Inference (BI) phylogenetic tree using PhyloSuite version 1.2.2 (Zhang D et al. 2020) with MAFFT version 7 (Katoh and Standley 2013) and MrBayes version 3.2.7 (Ronquist et al. 2012). Reference sequences of the subfamily Lamiinae published in formal publications were selected from the NCBI nucleotide database. The amino acid sequences of 13 PCGs in their mitogenomes were aligned using MAFFT with default parameters. According to Bayesian Information Criterion (BIC) scores, JTT (Jones-Taylor-Thornton) + F [State frequencies, dirichler (1.0,1.0,1.0,1.0)] + I [Proportion of invariable sites, uniformly distributed on the interval (0.00,1.00)] + G4 (gamma-distributed rate variation, four categories) model was selected as the best-fit partition model (edge-unlinked) using ModelFinder (Kalyaanamoorthy et al. 2017) for BI of amino acid sequences. In the BI analysis, two runs of 455,000 generations were conducted for each matrix, and the initial 25% were discarded as burn-in, which had the a same topology with an average standard deviation of split frequencies was 0.008362 (<0.01). The resulting phylogenetic tree is shown using FigTree 1.4.4 (https://github.com/rambaut/figtree/).

## Results

**Figure 2.**
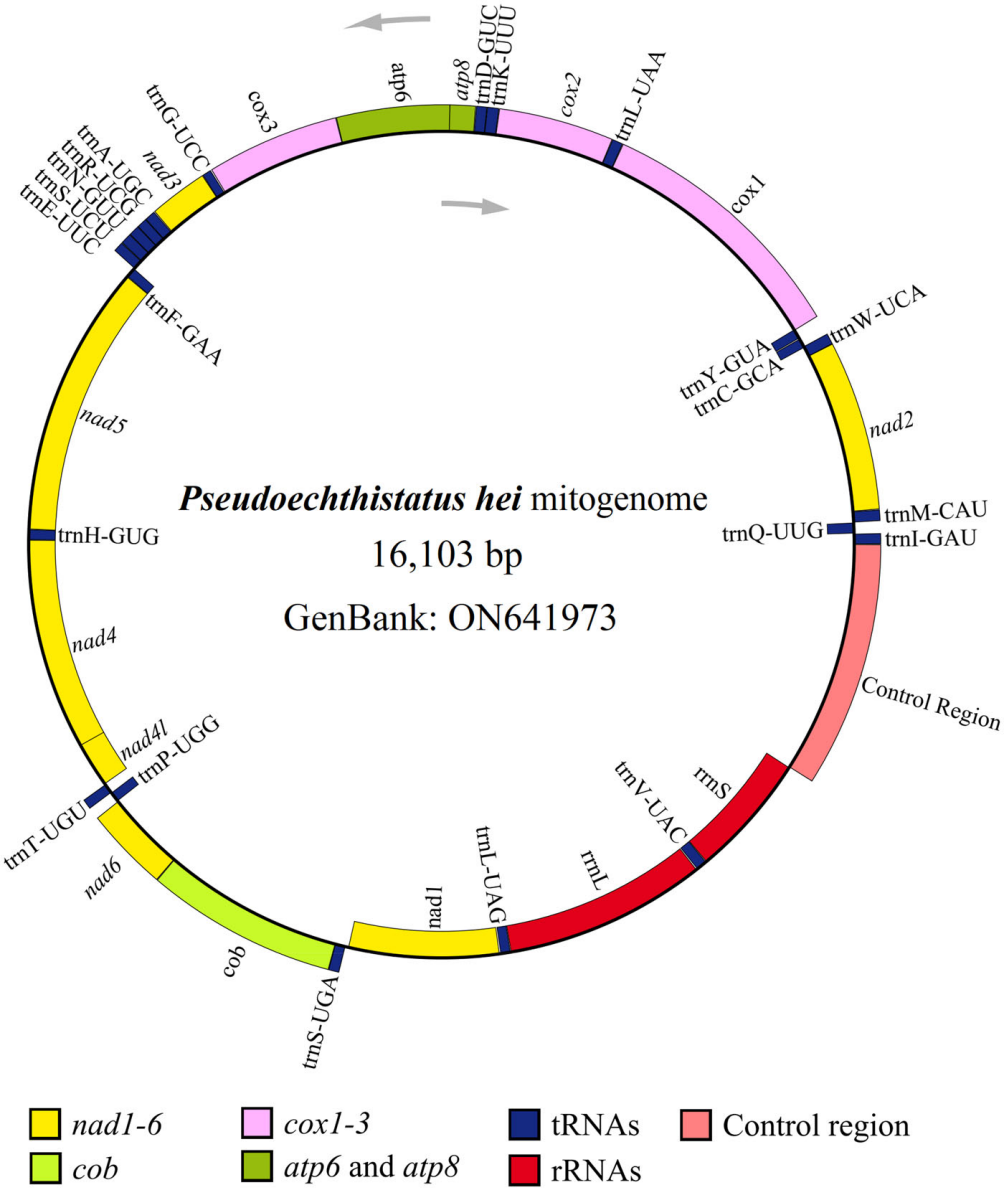
Mitogenome pattern map of *Pseudoechthistatus hei*. Grey arrows indicate the direction of gene transcription (5'→3'). Genes inside the black circle are coded in the majority strand (N-strand); genes outside the black circle are coded in the minority strand (J-strand).

We obtained approximately 43.88 Gb (97.76%) of clean high-quality data from 51.34 Gb of raw data. The circular mitogenome (GenBank accession number: ON641973.1) of *P. hei* was completely assembled and was 16,103 bp (nucleotide composition: 38.74% A, 36.96% T, 9.45% G, and 14.85% C) with 75.71% AT content. The AT- and GC-skew of the major strand of the mitogenome were estimated to be 0.0235 and −0.2224, respectively. The mitogenome of *P. hei* was comprised of 13 PCGs, 1 control region (CR), 22 tRNA genes, and two rRNA genes (Figure 2).

**Figure 3.**
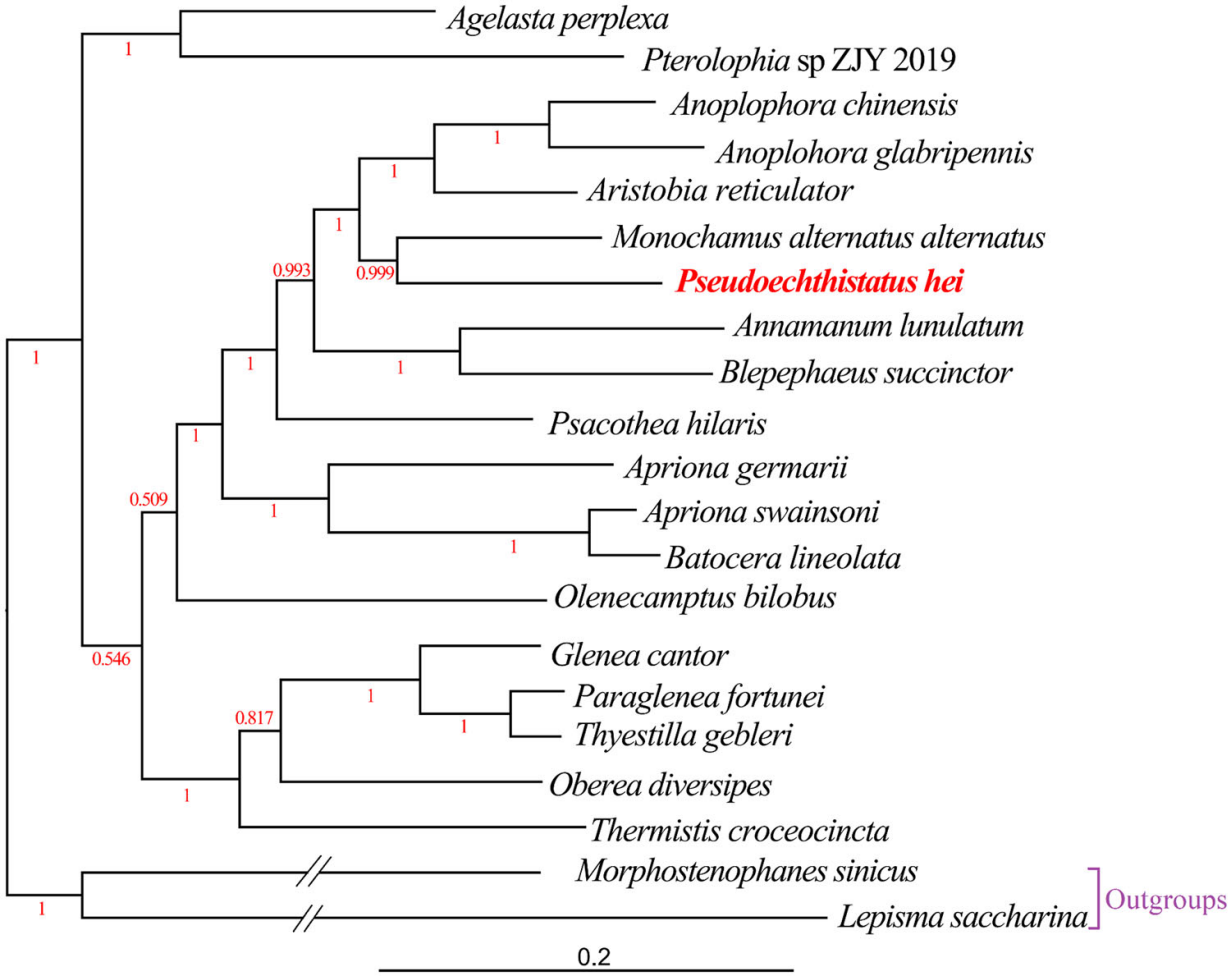
Bayesian Inference (BI) phylogenetic tree from amino acid sequences of 13 PCGs of 21 mitogenomes using MrBayes under the JTT + F + I + G4 model. BI posterior probability values were shown in red color. The complete mitogenome of *Pseudoechthistatus hei* (ON641973) determined in this study is indicated in red color. The branches of outgroups are depicted as half of their original branch length. The following sequences were used: *Apriona germari* MW858151 (Zhang Z-Y et al. 2021), *Paraglenea fortunei* MW858148 (Zhang Z-Y et al. 2021), *Annamanum lunulatum* MN356095 (Dai et al. 2020), *Aristobia reticulator* MK423971 (Behere et al. 2019), *Anoplophora chinensis* KT726932 (Li W et al. 2016), *Oberea diversipes* MN709785 (Tian and Wang 2020), *Agelasta perplexa* MW067123 (Li et al. 2021), *Olenecamptus bilobus* MT740324 (Dong et al. 2021), *Monochamus alternatus alternatus* MT547196 (Liao et al. 2020), *Batocera lineolata* JN986793 (Wang C et al. 2012), *Psacothea hilaris* FJ424074 (Ki-Gyoung et al. 2009), *Glenea cantor* MN044086 (Wang X et al. 2019), *Apriona swainsoni* KX184801 (Que et al. 2019), *Anoplophora glabripennis* DQ768215 (Fang et al. 2016), *Thyestilla gebleri* KY292221 (Yang et al. 2017), *Pterolophia* sp. ZJY-2019 MK863510 (Wang J et al. 2019), *Thermistis croceocincta* MK863511 (Wang J et al. 2019), *Blepephaeus succinctor* MK863507 (Wang J et al. 2019), *Morphostenophanes sinicus* MW853764 (Bai et al. 2021) and *Lepisma saccharina* MT108230 (Bai et al. 2020).

All 13 PCGs had a typical ATN (Met) start codon: two PCGs (*nad5* and *nad1*) initiated with an ATA start codon, five PCGs (*cox1*, *cox2*, *atp8*, *nad3*, and *nad6*) initiated with an ATT start codon, five PCGs (*atp6*, *cox3*, *nad4*, *nad4l*, and *cob*) initiated with an ATG start codon, and only one PCG (*nad2*) initiated with an ATC start codon. All 13 PCGs contained a typical TAN stop codon: two PCGs (*cob* and *nad1*) terminated with a TAG stop codon, seven PCGs (*nad2*, *atp8*, *atp6*, *nad3*, *nad5*, *nad4l*, and *nad6*) ended with a TAA stop codon, and four PCGs (*cox1*, *cox2*, *cox3*, and *nad4*) terminated with an incomplete stop codon (T), consisting of a codon that was completed by the addition of A nucleotides at the 3′ end of the encoded mRNA. The 22 tRNA ranged from 56 bp (trnC-GCA) to 72 bp (trnK-CUU) in length. The rrnL and rrnS genes were 1,276 and 785 bp in length, respectively. The CR, also an AT-rich region, was 1,460 bp in length, with an AT content of 80.55%, and was located between the rrnS and trnI-GAU genes. Based on the amino acid sequences of 13 PCGs from 21 mitogenomes, a phylogenetic tree was constructed using BI method (Figure 3), the structure of which was similar to that reported in previous studies (Liao et al. 2020). The results showed that *P. hei* was closely related to *Monochamus alternatus alternatus* with high support value (posterior probability value = 0.999).

## Discussion and conclusion

Although PCGs of the *P. hei* mitogenome only had typical ATN (Met) start codons, TTG, GTA and ATN were used as the start codons of *nad1* and *nad3* in mitogenomes of subfamily Lamiinae (Wang C-Y et al. 2013; Li F et al. 2016; Zhang Z-Y et al. 2021), which reflects the evolutionary diversity of the species and makes it difficult to determine the start and stop positions of PCGs. Taken together, the complete mitogenome of *P. hei* can contribute to the understanding of the mitogenome characteristics and determination of the phylogenetic position of the genus *Pseudoechthistatus*.

## Data Availability

The genome sequence data that support the findings of this study are openly available in GenBank of NCBI at https://www.ncbi.nlm.nih.gov under the accession no. ON641973.1. The associated BioProject, Bio-Sample, and SRA numbers are PRJNA857171, SAMN29620688, and SRR20067649, respectively.
